# A novel prognostic score (HAMP) for head and neck cancer patients with single and multiple SBRT‐treated lung metastases derived from retrospective analyses of survival outcome

**DOI:** 10.1002/hed.27913

**Published:** 2024-08-08

**Authors:** Samuel M. Vorbach, Thomas Seppi, Manuel P. Sarcletti, Siegfried Kollotzek, Julian Mangesius, Jens Lehmann, David Riedl, Martin J. Pointner, Matthias Santer, Daniel Dejaco, Meinhard Nevinny‐Stickel, Ute Ganswindt

**Affiliations:** ^1^ Department of Radiation Oncology Medical University of Innsbruck Innsbruck Austria; ^2^ Department of Psychiatry, Psychotherapy, Psychosomatics and Medical Psychology University Hospital of Psychiatry II, Medical University of Innsbruck Innsbruck Austria; ^3^ Department of Otorhinolaryngology – Head and Neck Surgery Medical University of Innsbruck Innsbruck Austria

**Keywords:** head and neck cancer, prognostic tool, pulmonary oligometastases, radiation oncology, stereotactic body radiotherapy

## Abstract

**Background:**

We report on the characterization and introduction of a novel prognostic score for patients undergoing stereotactic body radiotherapy (SBRT) for the treatment of single and multiple pulmonary metastases (PMs) derived from head and neck cancer (HNC).

**Methods:**

In this retrospective study, we examined selected factors associated with progression‐free survival (PFS) and overall survival (OS) among 59 patients with HNC treated with SBRT for a total of 118 PMs, between 2009 and 2023. Factors related to survival were included in the prognostic scoring system.

**Results:**

Prognostic factors including histology, age, number of metastases, and performance status at first SBRT were weighted differently depending on the strength of correlation to PFS and OS. Total prognostic scores (HAMP) ranged from 13 to 24 points, with a cut‐off total score of ≤18 scoring points for patients in a high‐risk (HR) subcohort, and of ≥19 scoring points for patients in a low‐risk group (LR). Median PFS (23.8 vs. 5.5 months, *p* < 0.001) and OS (61.3 vs. 16.4 months, *p* < 0.001) were significantly longer in the low‐risk group compared to the high‐risk group.

**Conclusion:**

The HAMP score might be a convenient tool to facilitate individualized treatment decisions and appropriate follow‐up. The accuracy and reliability of the score requires further evaluation in prospective studies.

## 1 INTRODUCTION

Head and neck cancer (HNC) ranks as the seventh most common tumor worldwide, accounting for more than 666 000 new cases and 325 000 deaths annually, with the overall incidence continuing to rise.^1^ Squamous cell carcinoma (SCC) is the most common histological type and the oral cavity is the most common site.^1^, ^2^ Even with combined treatment of surgery, radiotherapy, radiochemotherapy, and chemotherapy, the prognosis is poor with a 5‐year survival rate of approximately 50%.^3^

Distant metastases or locoregional failure occur in more than 40% of patients with HNC.^4^ Due to advances in treatment, such as volumetric‐modulated arc therapy (VMAT), together with improved staging and the increasing incidence of radiochemosensitive HPV‐related oropharyngeal cancer,^5^, ^6^ the long‐term locoregional control rate has reportedly increased towards 75%,^7^ thus making metastatic disease the main obstacle for improving overall survival (OS). At the time of diagnosis, approximately 3% of patients present distant metastases.^8^ However, as the disease progresses, up to 20%–30% of patients with HNC develop distant lesions.^4^, ^9^, ^10^, ^11^ The lung is the most common lesion site, accounting for up to 85% of all HNC‐derived distant metastases.^12^

Among local ablative treatments, stereotactic body radiotherapy (SBRT, also known as stereotactic ablative body radiotherapy, SABR) is an increasingly used noninvasive treatment option with excellent local control rates of over 90% for lesions in the lung. A biologically effective dose (BED) ≥ 100 Gy delivered on HNC‐derived pulmonary metastases is associated with a significantly better local control than less intensive radiation regimes.^13^ However, SBRT with elevated dose delivery is associated with higher pulmonary toxicity rates.^14^, ^15^ In a study of patients with early‐stage non‐small cell lung cancer, pulmonary toxicities ≥ grade 3 were associated with significant morbidity and mortality, particularly in patients with multiple comorbidities and impaired lung function.^16^ Nevertheless, SBRT is associated with good overall tolerability and safety, with most studies reporting toxicities ≥ grade 3 in only 5% of all patients.^17^, ^18^ Although SBRT has been endorsed by the National Comprehensive Cancer Network and the European Society for Medical Oncology as a suitable curative treatment option for pulmonary metastases in oligometastatic HNC, uncertainties persist in both patient selection and adequate choice of SBRT regimes.

As the risk of local recurrence increases with time after SBRT, local control of irradiated lung metastases is by nature more decisive for patients with a higher expected remaining lifetime, than for those with poor PFS and OS prognosis.

For patients at initial diagnosis of head and neck squamous cell carcinoma, the modified Glasgow Prognostic Score is currently being discussed for implementation as a prognostic tool.^19^ However, full evidence to guide decision making in the management of metastatic head and neck cancers (HNC) is lacking and no prognostic tool is available yet. Therefore, it would be advantageous to create a prognostic scoring tool that might assist in the management of increasingly occurring HNC‐derived pulmonary metastases. To determine patient prognosis and to help identify candidates who might best profit from SBRT, such tools have already been established for other sites of metastatic disease.^20^, ^21^

Therefore, in this initial retrospective study we aimed to develop a novel scoring system to predict PFS and OS of patients treated with SBRT for HNC‐derived pulmonary metastases. Ultimately, if this and subsequent cross‐validation studies are successful, we hoped that the newly introduced scoring system might help provide clinicians with additional support in the process of finding the most appropriate therapy for each individual patient. This includes selecting SBRT dosage and/or systemic therapy, as well as personalizing oncological follow‐up.

## 2 MATERIALS AND METHODS

### 2.1 Study sample

Fifty‐nine patients, with in total 118 histologically verified HNC‐derived pulmonary metastases and treated with SBRT at the Department of Radiation Oncology (Medical University of Innsbruck, Austria) between September 2009 and July 2023, were retrospectively identified and selected for analyses.

Demographic factors including age in years, sex, and smoking history were recorded at initial diagnosis, and again, at the time of SBRT. As a measure of patients' functional status and ability *for* self‐care at the time of SBRT, the Eastern Cooperative Oncology Group (ECOG) performance status was applied.^22^ Further characterization included data regarding the history, localization and treatment of the primary tumor, AJCC TNM classification, and p16 status for patients with oropharyngeal cancer if available.

Curative‐intent standard‐of‐care treatment of the primary HNC (see Table 1) as recommended by the institutional multidisciplinary tumor board was mandatory for inclusion in the retrospective study. In addition, all included patients were required to undergo CT‐guided biopsy for histological verification upon detection of lung lesions. All identified pulmonary metastases had to be treated with curative intent. Patients with local failure between initial treatment of the primary and the conclusion of their last SBRT, as well as patients with metastases to sites other than the lung were not included in this study.

**TABLE 1:** Patient, lesion, and treatment characteristics.

Characteristic		Value or *n* (%)
Total no. of patients		59
Total no. of metastases		118
Median age at start or SBRT (range)		65 (31–81)
Sex	Male	43 (72.9%)
	Female	16 (27.1%)
ECOG performance status (at the time of SBRT)	0	27 (45.8%)
	1	19 (32.2%)
	2	12 (20.3%)
	3	1 (1.7%)
Pack years at the time of first SBRT	Not available	1 (1.7%)
	0	5 (8.5%)
	<10	7 (11.9%)
	10–20	11 (18.6%)
	>20	35 (59.3%)
Primary tumor location	Hypopharynx	10 (16.9%)
	Larynx	12 (20.3%)
	Nasopharynx	2 (3.4%)
	Oral cavity	5 (8.5%)
	Oropharynx	23 (39.0%)
	Salivary gland	7 (11.9%)
Histology	SCC	51 (86.4%)
	Non‐SCC	8 (13.6%)
p16 status (oropharyngeal cancer only)	Not available	12 (52.2%)
	Positive	4 (17.4%)
	Negative	7 (30.4%)
AJCC tumor classification (valid at the time of diagnosis)	T1	8 (13.6%)
	T2	18 (30.5%)
	T3	15 (25.4%)
	T4	18 (30.5%)
AJCC nodal classification (valid at the time of diagnosis)	N0	20 (33.9%)
	N1	11 (18.6%)
	N2	26 (44.1%)
	N3	2 (3.4%)
UICC classification (valid at the time of diagnosis)	I	7 (11.9%)
	II	4 (6.8%)
	III	18 (30.5%)
	IV	30 (50.8%)
Primary treatment	Surgery	9 (15.3%)
	Surgery + radiotherapy	17 (28.8%)
	Surgery + radiochemotherapy	14 (23.7%)
	Radiotherapy	4 (6.8%)
	Radiochemotherapy	15 (25.4%)
Metastasis timing	Metachronous	51 (86.4%)
	Synchronous	8 (13.6%)
Courses of SBRT	1	38 (64.4%)
	2	18 (30.5%)
	3	3 (5.1%)
Treated lesions	Total number	118
	1. Lesion per SBRT	63 (=63) (53.4%)
	2. Lesions per SBRT	14 (=28) (23.7%)
	3. Lesions per SBRT	5 (=15) (12.7%)
	4. Lesions per SBRT	3 (=12) (10.2%)
Laterality	Left lower lobe	19 (16.1%)
	Left upper lobe	29 (24.6%)
	Right lower lobe	29 (24.6%)
	Right middle lobe	9 (7.6%)
	Right upper lobe	32 (27.1%)
Median internal gross target volume [cm^3^]/(range)		3.47 (0.19–49.61)
Dose, Gy/fraction and number of treated metastasis	60/10	18 (15.3%)
	48/4	3 (2.5%)
	48/8	1 (0.8%)
	48/6	21 (17.8%)
	45/3	75 (63.6%)
Number of metastases at the time of first SBRT	1	44 (74.6%)
	2	9 (15.2%)
	3	4 (6.8%)
	4	2 (3.4%)
Adjuvant systemic therapy	Yes	18 (30.5%)
	No	41 (69.5%)

Abbreviations: AJCC, American Joint Committee on Cancer; SCC, squamous cell carcinoma; UICC, Union for International Cancer Control.

The study was approved by the institutional review board of the Medical University of Innsbruck (EC No. approval: 1384/2022). All procedures conducted in this study involving human participants are in accordance with the ethical standards of the institutional review board as well as with the Helsinki Declaration (1964) and its later amendments or equivalent ethical standards.

### 2.2 Techniques of radiotherapy

The Elekta BodyFIX was used to immobilize patients in the supine position while a four‐dimensional CT scan tracked tumor movement over time. An internal gross target volume (IGTV) was defined to account for the effect of respiratory motion on the gross tumor volume (GTV). The internal gross target volume was expanded uniformly to create a planning target volume (PTV). To minimize radiation exposure to organs at risk, such as the lungs, spinal cord or the esophagus, their contours were delineated. Different dose strategies were used depending on the proximity of the lesion to critical organs: 60 Gy in 10 fractions (15.3%), 48 Gy in 4 fractions (2.5%), 48 Gy in 6 fractions (17.5%), 48 Gy in 8 fractions (0.8%), or 45 Gy in 3 fractions (63.6%).

Calculation of biologically effective doses for all prescription doses was performed using the linear quadratic model assuming an alpha/beta ratio of 10 (BED10). The corresponding dose prescription modalities included the 65% isodose, the 80% isodose, or the 100% isodose. Treatment planning was conducted using precisePLAN (Elekta AB, Stockholm, Sweden) until 2013 and subsequently with Pinnacle Software (latest version V14; Philips Medical, Fitchburg) until the study's conclusion. SBRT plans were generated by optimizing the target volume coverage and by minimizing dose on organs at risk. Coverage ranged from 0.88 to 1.00 (mean 0.98, 95% CI: 0.98–0.99). A conformity index (ratio of the absolute volume enclosed by the prescribed isodose to the absolute volume of the PTV) up to 1.6 was permitted (mean 1.46, 95% CI: 1.41–1.50).

Patients underwent treatment with three‐dimensional conformal radiation therapy (3D‐CRT) or VMAT using an Elekta Synergy linear accelerator until 2013, and a Versa HD linear accelerator thereafter (both from Elekta AB, Stockholm, Sweden). Daily cone beam CT scans were utilized to evaluate and, if needed, adjust patient positioning to ensure the inclusion of metastases in the planning target volume before each session.

### 2.3 Follow‐up

After completion of SBRT, follow‐up radiological imaging (CT scan, if recommended PET‐CT) was performed every 3 months for a total of 18 months and then every 6 months thereafter. Any equivocal findings were reviewed by a multidisciplinary tumor board, and if necessary, more frequent monitoring including a further CT scan was initiated. Two experienced radiologists classified tumor response according to Response Evaluation Criteria in Solid Tumors (RECIST) guidelines.^23^

Follow‐up was defined as the time between the end of SBRT and the last CT scan. Local control (LC) was defined as the number of passed days from the end of SBRT to local progression of the treated pulmonary metastases or to the date of last follow‐up, respectively. Progression‐free survival (PFS) was defined as the time from completion of SBRT to progression of treated metastases, or to the time of development of any new lesions, or to date of last follow‐up. Overall survival (OS) was defined as the time from completion of SBRT to either death from any cause or to the date of last follow‐up, respectively.

Toxicity was assessed by anamnesis at each follow‐up visit, physical examination, laboratory tests, and medical imaging, and it was graded according to the Common Terminology Criteria for Adverse Events (CTCAE version 3.0–5.0^24^).

### 2.4 Scoring and statistical analyses

The endpoints of this study were local control rate of treated metastases, progression‐free survival, and overall survival. SPSS Statistics (V26, IBM Corp., Armonk, NY) was used for analysis. Descriptive analysis summarized patient characteristics and treatment details. Spearman's rank correlation coefficient was used to test for correlation between the observed CTCAE graded toxicities during follow‐up and mean lung dose. The Kaplan–Meier method was used to calculate LC, PFS, and OS. Cox proportional hazards model was used for univariate and multivariate analyses of sex, age, histology, primary tumor location, UICC stage, primary treatment modality, number of pack years, and ECOG performance status, both assessed at the time of SBRT, metastasis timing, and number of metastasis to identify prognostic factors that were associated with PFS or OS. In deriving a prognostic score, we did not include variables associated with LC. For multivariable analysis, backward stepwise elimination of non‐significant factors was used. Significance was set at *p*‐values <0.05.

Consistent with previous studies of similar prognostic scoring systems,^25^, ^26^, ^27^ characteristics that showed a significant or near‐significant (*p* < 0.10) association with PFS and OS in the Cox regression analysis were the considered components of the presented scoring system. Since median follow‐up of the entire cohort was 37.2 months, the 3‐year overall survival rate was chosen for evaluating contributing prognostic factors. In accordance with previously published survival scoring systems,^25^, ^27^ the relative 3‐year OS rates were divided by 10, a transformation that converts survival percentages to single‐digit scores. The sum of the scoring points derived from the assessment of each prognostic factor represents the newly proposed prognostic score for outcome estimation of an individual patient.

Notably, the number of metastases included in the scoring analysis is limited to those lesions present at the time of first diagnosis of pulmonary metastases (*n* = 82; of a total of 118 pulmonary metastases). The scoring system presented is intended to predict the outcome of patients undergoing SBRT after their initial presentation of HNC‐derived pulmonary metastases. Therefore, by nature, metastases that appeared subsequently (*n* = 36) were not included in the analysis of this factor's potential in contributing to PFS and OS prediction.

Prognostic scores were divided into a low‐risk and a high‐risk group, using the mean value of the possible prognostic score as the threshold. Differences in the incidence of toxicities, that is, the occurrence or absence of toxicities, and differences in the administration of adjuvant systemic therapy between the low‐risk and the high‐risk group were assessed using the chi‐square test. Fisher's exact test was used to determine differences in the severity of the CTCAE graded toxicities between the low‐risk and the high‐risk cohort.

## 3 RESULTS

### 3.1 Sample

The present study includes 59 patients who underwent SBRT between September 2009 and July 2023, with a total of 118 lung lesions treated. The median follow‐up was 37.2 months (ranging from 3.1 to 171.6 months). Table 1 provides details of patient clinical data, lung lesions and treatment characteristics. The median age at the beginning of SBRT was 65.0 years (ranging from 31.4 to 81.4 years). The majority of patients were male (72.9%) and most of the patients (78.0%) had an ECOG performance status of 0–1 at the time of SBRT. The majority of patients had a history of smoking, with 59.3% having more than 20 pack‐years. Primary tumors were located in the hypopharynx, larynx, nasopharynx, oral cavity, salivary gland or in the oropharynx (10, 12, 2, 5, 7, and 23, respectively). The p16 status was available for 11 of the 24 patients (47.8%) with oropharyngeal tumors. Of these 11, 4 (36.4%) were p16 positive. At diagnosis, 33 patients (55.9%) were staged as T3‐T4 according to TNM classification, and 28 patients (47.5%) were staged as N2‐N3.

Primary treatment of HNC varied and included radiotherapy in all but nine patients, either alone or as part of a combined treatment approach. Of the 59 patients, 8 (13.6%) had synchronous metastatic disease, which was treated separately with SBRT in addition to primary therapy. The remaining 51 patients (86.4%) developed pulmonary metastases after completion of their primary treatment. Of these, 21 patients (35.6%) developed additional new lung metastases after conclusion of their first SBRT. These new lesions were again treated with SBRT and within this subgroup, three patients (5.1%) underwent SBRT 3 times for again newly appearing pulmonary metastases. None of the patients treated with SBRT received concomitant systemic therapy. However, 18 of the 59 patients received systemic therapy after their last SBRT due to further systemic progression later in the course of their disease.

At first SBRT (59 patients with a total of 82 pulmonary metastases), a single pulmonary metastasis was present and irradiated in 44 patients (74.6%). Two simultaneously present pulmonary metastases were treated in nine patients (15.2%), three pulmonary metastases in four patients (6.8%), and four pulmonary lesions were irradiated in two patients (3.4%).

The most common sites of metastasis were the left and right upper lobes, accounting for 49.2% of all pulmonary metastases. The median internal gross target volume was 3.47 cm^3^ (0.19–49.61 cm^3^). SBRT doses and fractionations were 45 Gy (3 fractions of 15 Gy; *n* = 75, 63.6%), 48 Gy (4 fractions of 12 Gy; *n* = 3, 2.5%; 6 fractions of 8 Gy; *n* = 21, 17.8%; 8 fractions of 6 Gy; *n* = 1, 0.8%), and 60 Gy (10 fractions of 6 Gy; *n* = 16, 17.4%). The median biologically effective dose was 112.5 Gy (76.8–112.5 Gy).

### 3.2 Outcome

Among all the patients who underwent SBRT, the overall 1‐year LC rate, PFS and OS were 95.9% (95% CI: 89.3–98.4%), 42.5% (29.6–54.8%), and 82.6% (70.1–90.2%), respectively, and the overall 2‐year LC rate, PFS and OS were 94.2% (86.4–97.6%), 30.3% (18.7–42.8%), and 56.6% (42.4–68.5%). Five years after SBRT, overall LC‐rate, PFS, and OS were 94.2% (86.4–97.6%), 17.3% (8.3–29.0%), and 30.4% (18.2–43.6%), respectively. Median OS was 36.5 months (range, 3.1–171.6 months; 95% CI: 19.3–55.2 months).

**FIGURE 1:**
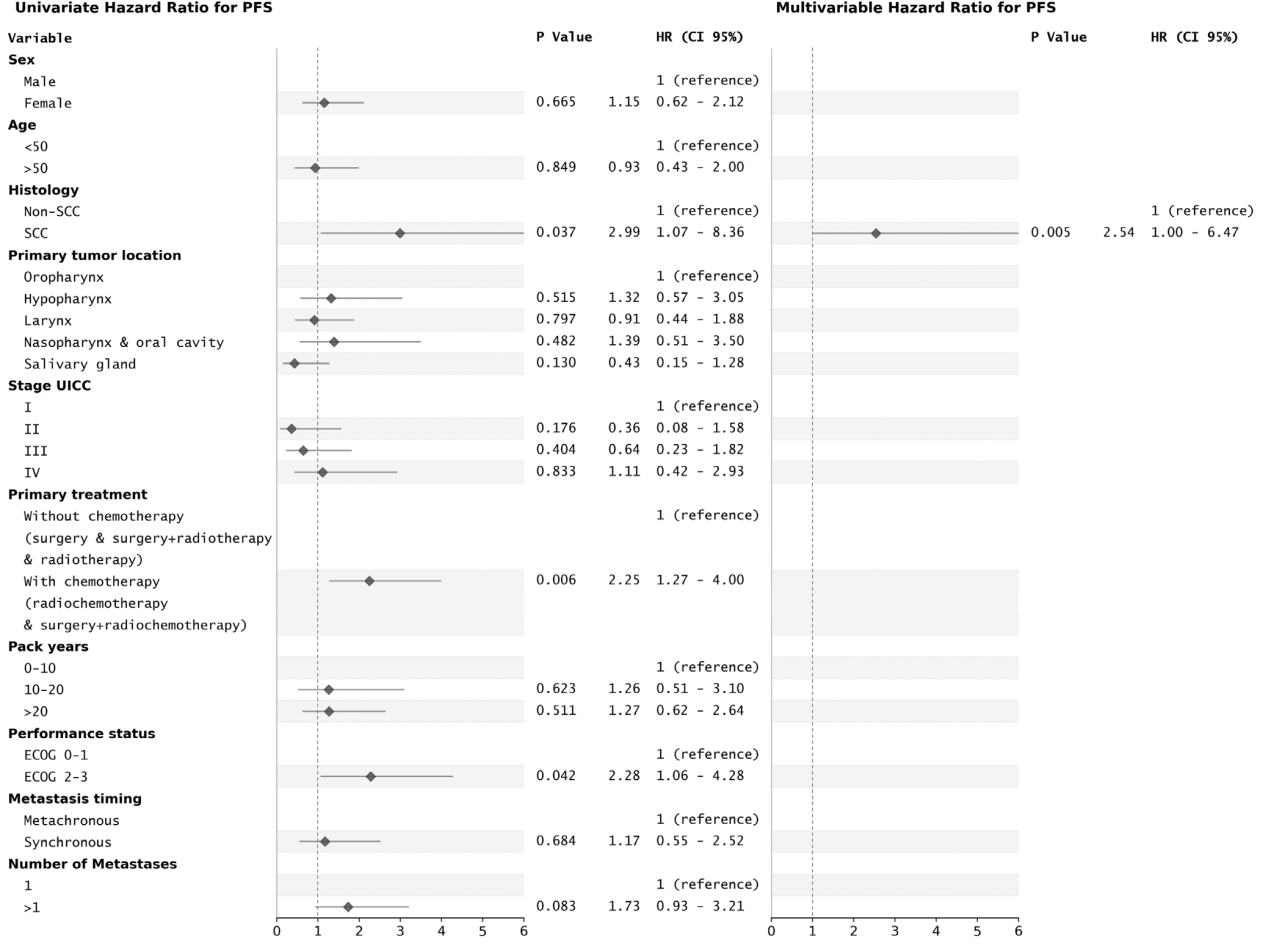
Univariate and multivariable analysis (Cox proportional hazards model) of factors related to progression‐free survival.

Prognostic factors significantly associated with reduced PFS in the univariate analysis were the histological subtype (SCC vs. non‐SCC; HR = 2.99, 95% CI: 1.07–8.36, *p* = 0.037; Figure 1), systemic co‐treatment of the primary tumor (radiochemotherapy and surgery followed by radiochemotherapy vs. surgery, surgery followed by radiotherapy, and radiotherapy; HR = 2.25, 95% CI: 1.27–4.00, *p* < 0.006), as well as poor patient performance status (ECOG 2–3 vs. ECOG 0–1; HR = 2.28, 95% CI: 1.06–4.28, *p* = 0.042).

In the multivariate analysis, histological subtype was the only significant prognostic factor associated with PFS (SCC vs. non‐SCC, HR = 2.54, 95% CI: 1.00–6.47, *p* = 0.005).

**FIGURE 2:**
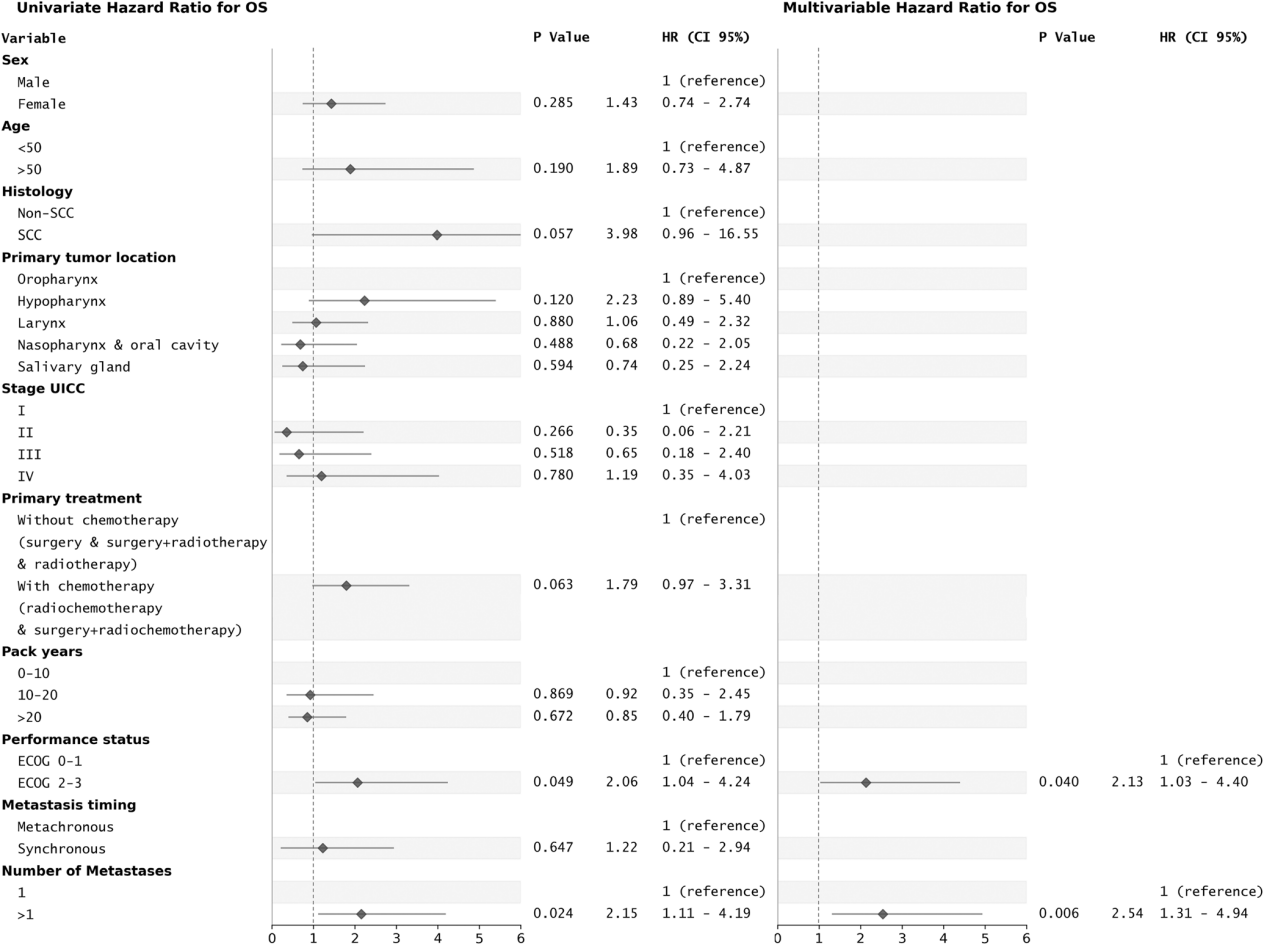
Univariate and multivariable analysis (Cox proportional hazards model) of factors related to overall survival.

In the univariate analysis of OS, more than one pulmonary metastasis (HR = 2.15, 95% CI: 1.11–4.19, *p* = 0.024) as well as poor performance status (ECOG 2–3 vs. ECOG 0–1, HR = 2.06, 95% CI: 1.04–4.24, *p* = 0.049; Figure 2) were evidenced to be factors significantly associated with reduced life expectancy.

More than one pulmonary metastasis (HR = 2.54, 95% CI: 1.31–4.94, *p* = 0.006) and poor patient performance status (ECOG 2–3 vs. ECOG 0–1, HR = 2.13, 95% CI: 1.03–4.40, *p* = 0.040) were confirmed in the multivariate analysis to be significantly associated with reduced OS.

Factors that were significantly or marginally significantly related to either PFS or OS in either univariate or multivariable analyses at a *p*‐value of <0.10 were included in the new prognostic score. The four factors included in the new scoring system were histological subtype of HNC, age of patients, number of metastases, and performance status, thus we choose the designated acronym “HAMP.” Although it was significantly associated with PFS and near‐significantly with OS, the primary treatment modality was not included as an independent factor in the prognostic scoring system, since therapy decision is mainly determined by the other already included factors, namely age, histological subtype, and patient performance status.

Median follow‐up time for the entire cohort was 37.2 months. The factor‐related 3‐year survival rates were divided by 10 to obtain the scoring points contributed by each prognostic component. Relative 3‐year survival rates and the corresponding scoring points are summarized in Table 2.

**TABLE 2:** Three‐year overall survival rates of the scoring factors and their corresponding scoring points.

Factor	Overall survival at 3 years (%)	Scoring points
Age (years)		
<50	55	6
>50	50	5
Histological subtype		
SCC	44	4
Non‐SCC	63	6
Performance status		
ECOG 0–1	56	6
ECOG ≥2	24	2
Metastases count at first SBRT		
1	61	6
≥2	24	2

Abbreviation: SCC, squamous cell carcinoma.

The total HAMP score of an individual patient was calculated by summing the scoring points derived from the assessment of each prognostic factor. Total scores ranged from 13 to 24 points. Patients were classified according to their HAMP score, thereby being assigned to a high‐risk or a low‐risk cohort. The mean value of the achievable score (=18.5 points) was used as the separating value for the cohort assignment (high‐risk cohort: 13–18 points, *N* = 27; low‐risk cohort: 19–24 points, *N* = 32).

For demonstration of HAMP score calculation, we offer the following example: SCC‐histology (=4 points), 65 years old (=5 points), three pulmonary metastases (=2 points), and performance status of ECOG 2 (=2 points), which results in a total HAMP score of 13 points, thus allocating this patient to the high‐risk group (see also Table 2).

**FIGURE 3:**
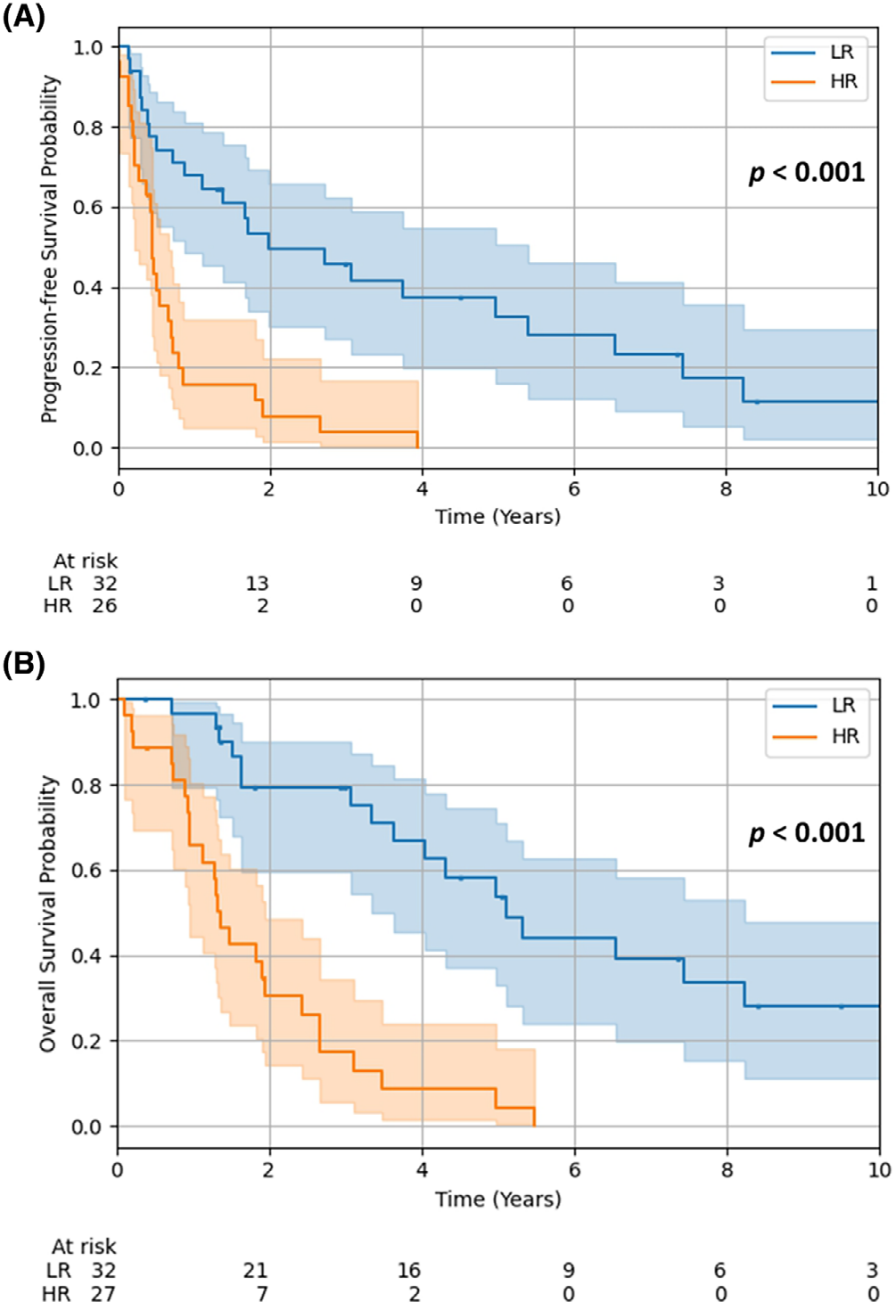
Kaplan–Meier curves showing (a) PFS and (b) OS for patients categorized into a high‐risk (*N* = 27) and a low‐risk group (*N* = 32) according to their individual HAMP score. *p*‐value was calculated using the log‐rank test. [Color figure can be viewed at wileyonlinelibrary.com]

Median PFS (23.8 months vs. 5.5 months, *p* < 0.001; Figure 3) and median OS (61.3 months vs. 16.4 months, *p* < 0.001; Figure 3) were significantly longer in the low‐risk group compared to the high‐risk group. The progression‐free survival rates at 1, 2, and 5 years were 64.7% (95% CI: 45.3–78.6%), 49.4% (95% CI: 30.4–65.9%), and 32.7% (95% CI: 15.9–50.6%) in the low‐risk group, as well as 15.7% (95% CI: 4.9–32.0%), 7.9% (95% CI: 1.4–22.1%), and 0.0% (95% CI: 0.0–0.0%) in the high‐risk group, respectively. Overall survival at 1, 2, and 5 years was 96.8% (95% CI: 79.2–99.5%), 79.3% (95% CI: 59.5–90.1%), and 53.9% (95% CI: 32.9–70.9%) in the low‐risk group, as well as 65.7% (95% CI: 44.4–80.5%), 30.4% (95% CI: 14.2–48.4%), and 4.3% (95% CI: 0.3–18.2%) in the high‐risk group, respectively.

Following treatment with SBRT, 18 of 59 patients (30.5%) received adjuvant systemic therapy due to further systemic progression during the later course of their disease. In the low‐risk group, adjuvant systemic therapy was administered in 7 out of 32 patients (21.9%), whereas in the high‐risk group, this was the case for 11 out of 27 patients (40.7%). There was no significant difference in the frequency of administration of adjuvant systemic therapy between the two risk groups (*χ* ^2^ [1, *N* = 59] = 2.49, *p* = 0.117).

### 3.3 Treatment‐related toxicities

The treatment was well tolerated, with 11 patients (18.6%) experiencing CTCAE grade 1 pulmonary toxicity and two patients (3.4%) developing grade 2 pneumonitis. There was a significant correlation between the mean lung dose and the observed rate of toxicities (Spearman's correlation coefficient: 0.47 (95% CI: 0.31–1.00); *p* < 0.001). No significant difference was detected in the overall rate and severity of toxicity between the low‐risk and the high‐risk group (overall rate: *χ* ^2^ [1, *N* = 59] = 0.48, *p* = 0.488, Fisher's exact test severity of toxicity: *p* = 0.185).

## 4 DISCUSSION

Noninvasive local therapies are known to be beneficial for patients with oligometastatic disease of varying origin.^28^ Recently, a small number of retrospective studies have shown promising OS data for patients also in the case of HNC‐derived pulmonary oligometastases treated with stereotactic ablative radiotherapy.^13^, ^29^, ^30^, ^31^, ^32^, ^33^, ^34^, ^35^, ^36^ Nevertheless, the evidence for treating pulmonary oligometastatic HNC with SBRT is limited so far, since several randomized trials are still ongoing or lack final reports (NCT03386357, NCT04862455, NCT03070366, NCT03862911, NCT03721341, NCT04989725, NCT03283605, NCT05136768, and NCT04498767). These trials, however, might further increase knowledge on the potential risks and benefits associated with multiple and/or repeated courses of SBRT of HNC‐derived pulmonary metastases, and equally important, independent prognostic factors for this specific cohort of patients might be validated or newly identified.

Prognostic scores intended to be useful tools to assist physicians and interdisciplinary tumor boards in their therapy decision‐making. Outcome‐estimating scores have been successfully introduced into clinical routine for several neoplastic diseases.^37^, ^38^, ^39^ However, less evidence is provided at present on specific patient characteristics and disease factors to reliably determine which patients would benefit the most from SBRT (and/or systemic treatment) of their HNC‐derived pulmonary metastases. The more favorable an individualized survival prognosis is, the more attention has to be addressed to local control and potential long‐term toxicity of adequately chosen therapy modalities. Thus, it is crucial to estimate the individual patient's life expectancy as reliably as possible.

For this purpose, the current analysis developed a novel survival‐predicting score specifically generated for patients with HNC‐derived oligometastatic pulmonary lesions. In our retrospective study, histological subtype, age, primary treatment modality, the number of pulmonary metastases and ECOG performance status were identified as factors significantly or marginally significantly related to PFS and/or OS. The factors significantly associated with OS (*p* < 0.05), that is, ECOG performance status and the number of pulmonary metastases, appeared to have a fairly strong discriminatory power regarding the categorization of patients into a high‐risk or a low‐risk group. This is reflected by the conversion of the associated survival percentages to score points; that is, ECOG performance status of 0–1, as well as one pulmonary lesion resulted in 6 score points, whereas ECOG performance status of 2–3, as well as more than one pulmonary lesion resulted in 2 score points. Histology, as the factor significantly associated with PFS (*p* < 0.05) and marginally significantly associated with OS (*p* = 0.057), contributed 6 points for the scoring of patients with tumors of other histology than squamous cell carcinoma, and 4 points for patients with squamous cell carcinoma. Finally, age was found to be associated marginally significantly with OS (*p* = 0.09) but not with PFS. Consequently, conversion of the associated survival percentages resulted in weaker discriminatory power, assigning 6 score points for patients younger than 50 years and 5 score points for patients older than 50 years, respectively.

In other retrospective reports of patients with metastatic HNC treated with SBRT, several parameters have been identified as potential prognostic factors for PFS and OS. These factors included, patient performance status, the size of the clinical target volume/planning target volume, presence of extra‐thoracic disease,^34^, ^35^ or the application of adjuvant systemic therapy.^34^ Other factors, such as sex, number of metastases, patient age, presence of bone or brain metastases, and the location of the primary tumor in the oral cavity are still being discussed as potential outcome predictors.^13^

For example, a retrospective study of Singh et al.^35^ reported that Karnofsky performance status, spinal disease and gross tumor volume were significant prognostic factors for OS after SBRT of squamous cell HNC metastases. Analogously to their finding regarding the role of Karnofsky performance status, we found that some levels of ECOG performance status were significantly related to OS in our cohort too. As gross tumor volume determination of lung lesions is usually not available prior to delineation by the treating radiation oncologist, we believe that this parameter cannot be included in a prognostic score at the time of treatment decision by the multidisciplinary tumor board. In addition, gross tumor volume definition is known to be subject to interobserver variation,^40^ whereas the number of pulmonary metastases included in our scoring system is a much more reliable parameter and easy to assess promptly. Also in contrast to our study, Singh et al. included patients with metastases located in several other areas than the lung (47% out of 98 lesions in total). By nature, their finding regarding spinal disease as a prognostic factor could not be validated in our study, since we only included patients with metastases located in the lung.

A similar retrospective study of Said et al.,^13^ which also included patients with HNC‐derived lesions located in regions other than the lung (50% out of 42 patients), only identified primary tumor location in the oral cavity as a significant factor in the univariate analysis of OS, but not in the multivariate analysis. The influences of histological subtype, age, and performance status were not investigated. In contrast to our results, Said et al. reported that the number of metastases were not a significant prognostic factor, which might be attributable to the heterogeneous sites of metastatic disease within their patient cohort.

Pasalic and colleagues^34^ retrospectively identified oligometastatic disease, adjuvant systemic therapy, and any smoking history as significant prognostic factors for OS after SBRT of exclusively HNC‐derived pulmonary metastases. In contrast to the results of Pasalic et al., the number of pack years did not turn out to be a prognostic factor in our cohort. However, 89.8% of our patients had a history of smoking, compared to the near‐equal distribution of smokers and non‐smokers in the study of Pasalic et al. The lack of evidence regarding smoking history as a significant factor in our cohort is most likely due to the very small sample size of non‐smokers (*N* = 5). In the univariate analysis of Pasalic et al., and in analogy to our findings also in their multivariate analysis, the histological subtype of SCC was associated with reduced OS. Pasalic et al. defined oligometastatic disease as the presence of up to three pulmonary lesions without active extra‐thoracic disease. The comparison with a “polymetastatic” group (of undefined extrathoracic lesion sites) revealed a better OS for the oligometastatic group. Unfortunately, this result is not comparable with our analysis, since we report on the outcome of pulmonary metastatic patients only. As a consequence, we categorized patients into two groups with one (*N* = 44) and with multiple pulmonary metastases (*N* = 15). The latter showed a statistically significant reduction in OS. Finally, we did not consider adjuvant systemic therapy as a potential prognostic factor, since it is by nature selectively used in patients with further systemic progression who are not candidates for further courses after their first SBRT. In other words, the need of adjuvant systemic therapy is evident in the retrospective view only, its application is unforeseeable before treatment decision‐making, and thus, it is not suitable as an independent parameter as part of the prognostic tool.

In our cohort, the only factor significantly associated with progression‐free survival in multivariable analysis was the histological subtype of HNC with SCC demonstrating worse prognosis. Said et al.^13^ reported on a cumulative planning target volume > 48 cc as an additional predictor for worse PFS, which however could not be confirmed in our retrospective analysis. At best in this regard, we found that more than one pulmonary metastasis in the univariate analysis was related to lower PFS, although not to a statistically significant extent.

Although not a component of the prognostic score, results on local control are presented for completeness. The LC rate of metastases of 95.9% at 1 year and of 94.2% at 2 years after SBRT is in best agreement with the findings of Pasalic et al.,^34^ who in their retrospective study reported an excellent 1‐year local control rate of 97.8% and 94.4% 2 years after SBRT.

We are aware of the limited sample size of our study. Nevertheless, our results may contribute to the growing evidence that SBRT is a very effective and well‐tolerated treatment option for patients with HNC‐derived lung metastases, even after repeated cycles of SBRT. A median OS of 36.5 months was achieved after the SBRT of pulmonary lesions and none of the patients was afflicted by a toxicity level higher than grade 2.

By applying the novel HAMP score, patients were categorized into two subcohorts of high‐ and low‐risk. The discriminatory power of the score‐based categorization was high, which is reflected in a significantly differing median OS of 61.3 months (95% CI: 44.5–78.1 months) in the low‐risk group and a median OS of 16.4 months (95% CI: 13.7–19.1 months) in the high‐risk group. Similar results could be achieved for PFS with a median PFS of 23.8 months (95% CI: 3.8–43.7 months) in the low‐risk group and a median PFS of 5.5 months (95% CI: 4.8–6.2 months) in the high‐risk group.

However, before applying the HAMP score, one should be aware about the retrospective nature of this study which requires caution in interpreting the findings. Although we present the outcome of one of the largest amount of SBRT‐treated HNC‐derived pulmonary metastases (*n* = 118) published to date, the still limited number of patients (*N* = 59) did not allow a statistically reasonable separation of the cohort into a training and a validation group. Furthermore, our retrospective analysis was limited by incomplete assessment of p16 status. In addition, potentially important covariates that were not investigated in this study such as comorbidities or socioeconomic status should be included in future investigations. The newly presented HAMP score still warrants validation by external datasets and in larger prospective cohorts.

## 5 CONCLUSIONS

The new HAMP score for predicting the survival outcome of patients receiving SBRT for the treatment of their pulmonary oligometastatic HNC is presented. It incorporates histology, age, number of metastases, and performance status as parameters to predict overall and progression‐free survival. The information required to calculate the score for an individual patient is part of the routinely collected basic clinical data. Thus, the score might be a suitable tool for oncologists to facilitate personalized treatment decisions as well as adequate follow‐up. However, further evaluation of the newly introduced HAMP score in prospective trials with regard to its accuracy and reliability is mandatory to promote its introduction into clinical routine.

## AUTHOR CONTRIBUTIONS

*Conception and design or analysis and interpretation of data*: S.M.V., D.D., M.S., M.P.S., M.N.‐S., U.G., T.S., S.K., M.J.P., and J.M. *Drafting of the manuscript or revising it for intellectual content*: S.M.V., D.R., J.L., T.S., and U.G.

## CONFLICT OF INTEREST STATEMENT

The authors declare no conflict of interest.

## ETHICS STATEMENT

The study was conducted in accordance with the Declaration of Helsinki, and approved by the Ethics Committee of the Medical University of Innsbruck (1384/2022). Patient consent was waived due to the retrospective study design.

## Data Availability

The data that support the findings of this study are available from the corresponding author upon reasonable request.
